# (Ferrocenyl­thio­phospho­nato-κ*S*)(triphenyl­phosphane-κ*P*)gold(I) dichloro­methane monosolvate

**DOI:** 10.1107/S1600536810039255

**Published:** 2010-10-09

**Authors:** Hendriette van der Walt, Alfred Muller, Richard J. Staples, Werner E. Van Zyl

**Affiliations:** a Research Centre in Synthesis and Catalysis, Department of Chemistry, University of Johannesburg (APK Campus), PO Box 524, Auckland Park, Johannesburg 2006, South Africa; b Department of Chemistry, Michigan State University, East Lansing, MI 48824-1322, USA; c School of Chemistry, University of KwaZulu-Natal, Westville Campus, Private Bag X54001, Durban 4000, South Africa

## Abstract

In the title compound, [AuFe(C_5_H_5_)(C_5_H_5_O_2_PS)(C_18_H_15_P)]·CH_2_Cl_2_, the two-coordinate gold(I) atom shows a slightly distorted linear arrangement, with a P—Au—S bond angle of 176.81 (6)°. The difference in P=O and P—O(H) bond lengths, which are 1.503 (6) and 1.541 (5) Å, respectively, implies there is apparently no delocalization between the P—O bonds, and the proton appears to be localized on one O atom only. In the crystal structure, inter­molecular O—H⋯O hydrogen bonds link dinuclear mol­ecules into chains propagated in the [010] direction. The dichloro­methane solvent mol­ecule was disordered between two positions in a 0.63 (3):0.37 (3) ratio.

## Related literature

For information on dithio­phospho­nate complexes of Group 11 metals, see: Van Zyl (2010 ▶). For the synthesis of dithio­phospho­nate salt derivatives, see: Van Zyl & Fackler (2000 ▶). For gold complexes with thio­phosphoryl-based ligands, see: Crespo *et al.* (2004 ▶). For gold complexes with dithio­phosphate phosphine gold(I) complexes, see: Preisenberger *et al.* (1998 ▶). For the synthesis of ferrocenyl (Fc) dimers of the type [PS_2_(Fc)]_2_, see: Foreman *et al.* (1996 ▶). For general background, see: Allen (2002 ▶). 
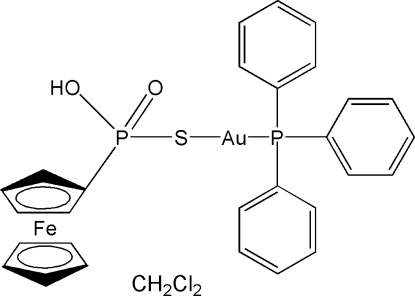


## Experimental

### 

#### Crystal data

[AuFe(C_5_H_5_)(C_5_H_5_O_2_PS)(C_18_H_15_P)]·CH_2_Cl_2_
*M* *_r_* = 825.22
Monoclinic, 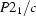

*a* = 15.122 (3) Å
*b* = 9.3157 (18) Å
*c* = 22.581 (3) Å
β = 112.831 (10)°
*V* = 2931.8 (9) Å^3^
*Z* = 4
Mo *K*α radiation
μ = 5.88 mm^−1^
*T* = 173 K
0.20 × 0.18 × 0.16 mm

#### Data collection

Bruker APEXII CCD diffractometer
Absorption correction: multi-scan (*SADABS*; Bruker, 1998 ▶) *T*_min_ = 0.386, *T*_max_ = 0.453
13808 measured reflections
4924 independent reflections
4348 reflections with *I* > 2σ(*I*)
*R*_int_ = 0.112

#### Refinement

*R*[*F* ^2^ > 2σ(*F* ^2^)] = 0.051
*wR*(*F* ^2^) = 0.153
*S* = 1.10
4924 reflections
375 parameters
18 restraints
H atoms treated by a mixture of independent and constrained refinement
Δρ_max_ = 2.14 e Å^−3^
Δρ_min_ = −1.63 e Å^−3^

### 

Data collection: *APEX2* (Bruker 2006 ▶); cell refinement: *SAINT-Plus* (Bruker, 2001 ▶); data reduction: *SAINT-Plus*; program(s) used to solve structure: *SIR2002* (Burla *et al.*, 2003 ▶); program(s) used to refine structure: *SHELXL97* (Sheldrick, 2008 ▶); molecular graphics: *DIAMOND* (Brandenburg & Berndt, 2001 ▶); software used to prepare material for publication: *WinGX* (Farrugia, 1999 ▶).

## Supplementary Material

Crystal structure: contains datablocks global, I. DOI: 10.1107/S1600536810039255/cv2770sup1.cif

Structure factors: contains datablocks I. DOI: 10.1107/S1600536810039255/cv2770Isup2.hkl

Additional supplementary materials:  crystallographic information; 3D view; checkCIF report

## Figures and Tables

**Table 1:** Hydrogen-bond geometry (Å, °)

*D*—H⋯*A*	*D*—H	H⋯*A*	*D*⋯*A*	*D*—H⋯*A*
O2—H1⋯O1^i^	1.11 (11)	1.33 (11)	2.432 (7)	173 (9)

Symmetry code: (i) 
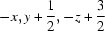
.

**Fig. 1.**
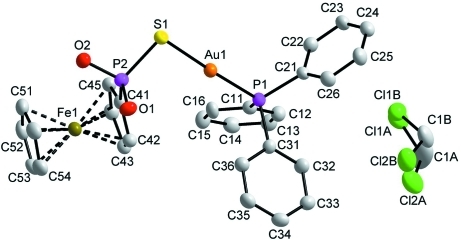
View of (I) (30% probability displacement ellipsoids). Hydrogen atoms omitted for clarity.

**Fig. 2.**
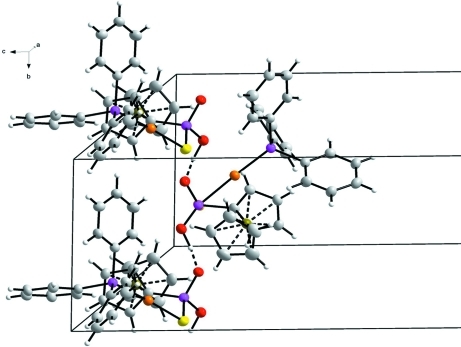
Packing diagram of (I) showing the infinite hydrogen bonding interactions (dashed lines) along the b axis. Solvent molecules omitted for clarity.

## supplementary crystallographic information

### Comment

The dithiophosphonato dianion salt, (NH_4_)_2_[S_2_P(Fc)OCH_2_CH_2_O(Fc)PS_2_] (Fc = ferrocenyl) was used in this study. It was obtained from the reaction between the dimer [PS_2_(Fc)]_2_ (Foreman *et al.*, 1996) and ethanediol, which formed a diacid. The diacid could be readily deprotonated by ammonia gas. The salt was then reacted with [Au(PPh_3_)Cl] to yield the complex [(PPh_3_)AuS_2_(Fc)P(OC_2_H_4_O)P(Fc)S_2_Au(PPh_3_)] (I). The complex was fully characterized spectroscopically (see Experimental). During work-up for crystal growth, however, the ligand became oxidized, presumably as a result of water present in the solvent dichloromethane. This resulted in substitution of a terminal P═S bond with a P═O bond, a reaction that can readily occur with oxophilic phosphorus(V) in the presence of moisture. Additionally, it resulted in C—O bond cleavage to be replaced with H—O bond formation. A precise mechanism for the reaction is not proposed. The title compound I (see Fig 1) crystalizes in the *P*2_1_/*c* space group with molecules lying on general positions in the unit cell. All geometrical data for the compound are within the normal limits (Allen, 2002). In the crystal packing, there is a hydrogen-bonding network along the *b* axis between the P═ O···H—O—P moieties (see Fig 2 and Table 1).

### Experimental

The complex (I) was obtained as an oxidized product. The initial reaction was between the (NH_4_)_2_[S_2_P(Fc)OCH_2_CH_2_O(Fc)PS_2_] salt (Fc = ferrocenyl) (0.122 g, 0.206 mmol) and [Au(PPh_3_)Cl] (0.200 g, 0.413 mmol) in a THF solution. The NH_4_Cl was filtered off and the filtrate solvent removed under reduced pressure yielding a yellow colored powder. Yield: 0.127 g (41%). *M*.p.: 128°C. ^1^H-NMR (CDCl_3_) δ: 7.54 – 7.45 (m, 30H, PPh_3_); 4.65 – 4.22 (d, 4H, Fc); 4.53 – 4.50 (d, 4H, Fc); 4.30 (s, 10H, Fc); 4.36 – 4.31 (t, 2H, CH_2_); 3.99 – 3.93 (m, 2H, CH_2_). ^31^P-NMR δ: 106.14 and 106.03 (d, 2P, P—S); 37.82 (s, 2P, PPh_3_). ESI-MS: m/*z* 1539 (95%) for [(PPh_3_)AuS_2_(Fc)P(OC_2_H_4_O)P(Fc)S_2_Au(PPh_3_)].

### Refinement

The aromatic H atoms were placed in geometrically idealized positions (C—H = 0.95 Å) and constrained to ride on their parent atoms with *U*_iso_(H) = 1.2*U*_eq_(C). The hydroxyl H was located in a Fourier difference map and refined isotropically. The disorder of the solvent molecule was refined over two positions that adds to unity. The final refinement shows a ratio of 37:63 for the two components. The neccesary bond and *U^ij^* restraints were applied to keep the refinement stable.

### Crystal data

**Table:**

[AuFe(C_5_H_5_)(C_5_H_5_O_2_PS)(C_18_H_15_P)]·CH_2_Cl_2_	*F*(000) = 1608
*M**_r_* = 825.22	*D*_x_ = 1.87 Mg m^−^^3^
Monoclinic, *P*2_1_/*c*	Mo *K*α radiation, λ = 0.71073 Å
Hall symbol: -P 2ybc	Cell parameters from 9574 reflections
*a* = 15.122 (3) Å	θ = 2.2–27.9°
*b* = 9.3157 (18) Å	µ = 5.88 mm^−^^1^
*c* = 22.581 (3) Å	*T* = 173 K
β = 112.831 (10)°	Block, orange-brown
*V* = 2931.8 (9) Å^3^	0.2 × 0.18 × 0.16 mm
*Z* = 4	

### Data collection

**Table:**

Bruker CCD diffractometer	4348 reflections with *I* > 2σ(*I*)
graphite	*R*_int_ = 0.112
φ scans	θ_max_ = 25°, θ_min_ = 1.5°
Absorption correction: multi-scan (*SADABS*; Bruker, 1998)	*h* = −16→17
*T*_min_ = 0.386, *T*_max_ = 0.453	*k* = −11→11
13808 measured reflections	*l* = −26→20
4924 independent reflections	

### Refinement

**Table:**

Refinement on *F*^2^	Primary atom site location: structure-invariant direct methods
Least-squares matrix: full	Secondary atom site location: difference Fourier map
*R*[*F*^2^ > 2σ(*F*^2^)] = 0.051	Hydrogen site location: inferred from neighbouring sites
*wR*(*F*^2^) = 0.153	H atoms treated by a mixture of independent and constrained refinement
*S* = 1.10	*w* = 1/[σ^2^(*F*_o_^2^) + (0.095*P*)^2^] where *P* = (*F*_o_^2^ + 2*F*_c_^2^)/3
4924 reflections	(Δ/σ)_max_ = 0.001
375 parameters	Δρ_max_ = 2.14 e Å^−^^3^
18 restraints	Δρ_min_ = −1.63 e Å^−^^3^

### Special details

**Table:**

Experimental. The intensity data was collected on a Bruker CCD based diffractometer equipped with an Oxford Cryostream low-temperature apparatus operating at 173 K diffractometer using an exposure time of 30 s/frame. A total of 1276 frames were collected with a frame width of 0.5° covering up to θ = 28.63% with 86.5% completeness accomplished. Due to decomposition and the weak diffracting nature of the title compound, refinement of data was restricted to θ = 25.0% to reach a completeness of 95.5%.
Geometry. All e.s.d.'s (except the e.s.d. in the dihedral angle between two l.s. planes) are estimated using the full covariance matrix. The cell e.s.d.'s are taken into account individually in the estimation of e.s.d.'s in distances, angles and torsion angles; correlations between e.s.d.'s in cell parameters are only used when they are defined by crystal symmetry. An approximate (isotropic) treatment of cell e.s.d.'s is used for estimating e.s.d.'s involving l.s. planes.
Refinement. Refinement of *F*^2^ against ALL reflections. The weighted *R*-factor *wR* and goodness of fit *S* are based on *F*^2^, conventional *R*-factors *R* are based on *F*, with *F* set to zero for negative *F*^2^. The threshold expression of *F*^2^ > σ(*F*^2^) is used only for calculating *R*-factors(gt) *etc*. and is not relevant to the choice of reflections for refinement. *R*-factors based on *F*^2^ are statistically about twice as large as those based on *F*, and *R*- factors based on ALL data will be even larger.

### Fractional atomic coordinates and isotropic or equivalent isotropic displacement parameters (Å^2^)

**Table:**

	*x*	*y*	*z*	*U*_iso_*/*U*_eq_	Occ. (<1)
Au1	0.227778 (18)	0.21410 (3)	0.709726 (13)	0.04416 (16)	
Fe1	−0.15433 (8)	0.30798 (11)	0.60212 (6)	0.0463 (3)	
S1	0.17770 (13)	0.35983 (19)	0.77340 (10)	0.0499 (4)	
P1	0.28030 (12)	0.08231 (19)	0.64580 (9)	0.0407 (4)	
P2	0.03846 (15)	0.29088 (17)	0.74410 (11)	0.0443 (5)	
O1	0.0279 (4)	0.1480 (6)	0.7716 (3)	0.0528 (13)	
O2	−0.0137 (4)	0.4034 (6)	0.7688 (3)	0.0502 (12)	
C11	0.2290 (5)	0.1421 (7)	0.5625 (3)	0.0439 (15)	
C12	0.2703 (5)	0.1110 (8)	0.5188 (4)	0.0515 (17)	
H12	0.3305	0.0634	0.5326	0.062*	
C13	0.2237 (6)	0.1493 (8)	0.4552 (4)	0.0544 (18)	
H13	0.2521	0.1274	0.4254	0.065*	
C14	0.1361 (7)	0.2190 (8)	0.4340 (5)	0.058 (2)	
H14	0.1054	0.2476	0.3904	0.069*	
C15	0.0933 (7)	0.2469 (10)	0.4779 (4)	0.058 (2)	
H15	0.0317	0.2903	0.4639	0.069*	
C16	0.1411 (6)	0.2112 (7)	0.5408 (4)	0.0514 (19)	
H16	0.1132	0.2344	0.5707	0.062*	
C21	0.4100 (5)	0.0891 (7)	0.6701 (3)	0.0435 (15)	
C22	0.4526 (6)	0.2000 (8)	0.6510 (4)	0.0516 (19)	
H22	0.4133	0.2703	0.6224	0.062*	
C23	0.5507 (6)	0.2118 (7)	0.6723 (5)	0.055 (2)	
H23	0.579	0.2881	0.6579	0.067*	
C24	0.6073 (5)	0.1112 (9)	0.7150 (4)	0.0542 (18)	
H24	0.6751	0.118	0.7295	0.065*	
C25	0.5671 (5)	0.0007 (9)	0.7369 (4)	0.063 (2)	
H25	0.6069	−0.0661	0.7674	0.075*	
C26	0.4683 (5)	−0.0116 (8)	0.7141 (4)	0.0522 (17)	
H26	0.44	−0.0884	0.7282	0.063*	
C31	0.2488 (5)	−0.1068 (7)	0.6423 (3)	0.0428 (15)	
C32	0.2881 (6)	−0.2077 (7)	0.6148 (4)	0.0499 (19)	
H32	0.3353	−0.18	0.599	0.06*	
C33	0.2582 (6)	−0.3508 (8)	0.6105 (4)	0.0540 (18)	
H33	0.2868	−0.4213	0.5931	0.065*	
C34	0.1871 (5)	−0.3890 (9)	0.6314 (4)	0.0559 (19)	
H34	0.1657	−0.4858	0.6274	0.067*	
C35	0.1471 (6)	−0.2889 (8)	0.6581 (5)	0.058 (2)	
H35	0.0991	−0.3167	0.6732	0.07*	
C36	0.1766 (5)	−0.1480 (8)	0.6628 (4)	0.0505 (17)	
H36	0.1476	−0.0784	0.6803	0.061*	
C41	−0.0125 (5)	0.2862 (7)	0.6583 (4)	0.0452 (18)	
C42	−0.0462 (5)	0.1626 (8)	0.6188 (4)	0.0476 (17)	
H42	−0.0448	0.0669	0.6336	0.057*	
C43	−0.0826 (6)	0.2068 (8)	0.5530 (4)	0.0490 (18)	
H43	−0.1094	0.1461	0.5166	0.059*	
C44	−0.0713 (5)	0.3575 (8)	0.5522 (4)	0.0504 (17)	
H44	−0.0896	0.4162	0.5149	0.061*	
C45	−0.0278 (5)	0.4060 (8)	0.6169 (4)	0.0467 (16)	
H45	−0.0117	0.5027	0.6302	0.056*	
C51	−0.2328 (6)	0.4488 (10)	0.6316 (5)	0.070 (2)	
H51	−0.2125	0.5385	0.6524	0.083*	
C52	−0.2765 (6)	0.4242 (12)	0.5630 (5)	0.074 (3)	
H52	−0.2884	0.4942	0.5302	0.089*	
C53	−0.2981 (6)	0.2771 (10)	0.5542 (5)	0.069 (3)	
H53	−0.329	0.2308	0.5139	0.082*	
C54	−0.2672 (7)	0.2106 (10)	0.6135 (6)	0.066 (3)	
H54	−0.2727	0.1112	0.6207	0.08*	
C55	−0.2263 (6)	0.3149 (10)	0.6614 (5)	0.060 (2)	
H55	−0.1988	0.2976	0.7064	0.072*	
H1	−0.020 (7)	0.512 (12)	0.747 (4)	0.09 (3)*	
C1A	0.539 (6)	−0.310 (3)	0.556 (4)	0.18 (4)	0.37 (3)
H1A1	0.5102	−0.3393	0.51	0.217*	0.37 (3)
H1A2	0.6083	−0.3287	0.5704	0.217*	0.37 (3)
Cl1A	0.528 (3)	−0.134 (3)	0.5549 (15)	0.094 (5)	0.37 (3)
Cl2A	0.4991 (7)	−0.427 (2)	0.5939 (16)	0.117 (8)	0.37 (3)
C1B	0.5793 (14)	−0.284 (2)	0.6005 (13)	0.093 (8)	0.63 (3)
H1B1	0.5783	−0.3424	0.5636	0.112*	0.63 (3)
H1B2	0.6472	−0.2746	0.6303	0.112*	0.63 (3)
Cl1B	0.5391 (15)	−0.1218 (16)	0.5733 (8)	0.081 (3)	0.63 (3)
Cl2B	0.5235 (9)	−0.3745 (11)	0.6373 (9)	0.106 (5)	0.63 (3)

### Atomic displacement parameters (Å^2^)

**Table:**

	*U*^11^	*U*^22^	*U*^33^	*U*^12^	*U*^13^	*U*^23^
Au1	0.0426 (2)	0.0476 (2)	0.0452 (2)	−0.00013 (9)	0.02019 (16)	−0.00134 (11)
Fe1	0.0409 (6)	0.0511 (5)	0.0486 (7)	−0.0013 (4)	0.0191 (5)	−0.0046 (5)
S1	0.0459 (9)	0.0530 (10)	0.0529 (11)	−0.0054 (7)	0.0215 (8)	−0.0097 (9)
P1	0.0396 (9)	0.0443 (8)	0.0418 (10)	−0.0003 (7)	0.0196 (7)	0.0001 (8)
P2	0.0471 (11)	0.0430 (10)	0.0443 (11)	−0.0016 (7)	0.0192 (9)	−0.0016 (8)
O1	0.072 (3)	0.042 (3)	0.048 (3)	−0.005 (2)	0.028 (3)	0.001 (2)
O2	0.056 (3)	0.050 (3)	0.054 (3)	0.000 (2)	0.031 (3)	−0.002 (2)
C11	0.045 (4)	0.047 (4)	0.041 (4)	−0.003 (3)	0.018 (3)	−0.001 (3)
C12	0.049 (4)	0.054 (4)	0.051 (4)	0.003 (3)	0.019 (3)	0.000 (4)
C13	0.065 (5)	0.057 (4)	0.046 (4)	0.003 (4)	0.027 (4)	0.008 (4)
C14	0.060 (5)	0.059 (5)	0.047 (5)	−0.001 (3)	0.013 (4)	0.009 (4)
C15	0.060 (5)	0.064 (4)	0.050 (5)	0.013 (4)	0.022 (4)	0.010 (4)
C16	0.042 (4)	0.061 (5)	0.059 (5)	0.005 (3)	0.028 (4)	0.008 (4)
C21	0.036 (3)	0.048 (3)	0.047 (4)	−0.005 (3)	0.017 (3)	−0.009 (3)
C22	0.053 (5)	0.049 (4)	0.053 (5)	0.003 (3)	0.021 (4)	0.002 (3)
C23	0.046 (4)	0.056 (5)	0.070 (6)	−0.006 (3)	0.028 (4)	−0.004 (4)
C24	0.045 (4)	0.065 (4)	0.054 (5)	−0.003 (3)	0.020 (3)	−0.007 (4)
C25	0.046 (4)	0.068 (5)	0.071 (5)	0.009 (4)	0.019 (4)	0.007 (4)
C26	0.051 (4)	0.056 (4)	0.053 (5)	−0.003 (3)	0.023 (3)	0.006 (4)
C31	0.044 (4)	0.043 (3)	0.044 (4)	0.003 (3)	0.020 (3)	0.002 (3)
C32	0.056 (5)	0.053 (4)	0.050 (5)	−0.004 (3)	0.031 (4)	0.000 (3)
C33	0.056 (4)	0.047 (4)	0.059 (5)	0.000 (3)	0.022 (4)	−0.002 (4)
C34	0.062 (5)	0.053 (4)	0.054 (5)	−0.014 (3)	0.024 (4)	−0.002 (4)
C35	0.050 (5)	0.061 (5)	0.069 (6)	−0.009 (3)	0.028 (4)	0.001 (4)
C36	0.050 (4)	0.050 (4)	0.054 (5)	−0.002 (3)	0.024 (3)	0.001 (4)
C41	0.040 (4)	0.051 (4)	0.049 (5)	0.001 (3)	0.023 (3)	−0.004 (3)
C42	0.044 (4)	0.049 (4)	0.050 (4)	−0.003 (3)	0.019 (3)	−0.005 (4)
C43	0.045 (4)	0.060 (5)	0.042 (4)	0.000 (3)	0.017 (3)	−0.007 (3)
C44	0.049 (4)	0.060 (4)	0.045 (4)	0.003 (3)	0.021 (3)	0.009 (4)
C45	0.047 (4)	0.047 (4)	0.052 (4)	−0.003 (3)	0.026 (3)	0.000 (3)
C51	0.048 (4)	0.072 (5)	0.092 (7)	0.004 (4)	0.032 (5)	−0.020 (5)
C52	0.047 (4)	0.090 (6)	0.081 (7)	0.017 (4)	0.021 (4)	0.000 (6)
C53	0.039 (5)	0.094 (7)	0.072 (7)	−0.013 (4)	0.020 (4)	−0.030 (5)
C54	0.053 (5)	0.075 (6)	0.082 (7)	−0.009 (4)	0.039 (5)	−0.008 (5)
C55	0.054 (5)	0.082 (5)	0.055 (5)	−0.006 (4)	0.032 (4)	−0.006 (5)
C1A	0.19 (7)	0.101 (13)	0.34 (11)	0.00 (4)	0.20 (8)	0.00 (4)
Cl1A	0.070 (6)	0.100 (7)	0.098 (13)	−0.005 (6)	0.016 (10)	0.011 (8)
Cl2A	0.077 (5)	0.108 (9)	0.147 (17)	0.009 (5)	0.020 (7)	0.040 (12)
C1B	0.051 (10)	0.097 (12)	0.13 (2)	0.014 (8)	0.029 (11)	0.029 (13)
Cl1B	0.076 (6)	0.079 (3)	0.084 (7)	0.002 (3)	0.026 (6)	−0.008 (4)
Cl2B	0.098 (5)	0.090 (4)	0.149 (11)	0.016 (4)	0.068 (7)	0.034 (5)

### Geometric parameters (Å, °)

**Table:**

Au1—P1	2.2609 (18)	C32—H32	0.95
Au1—S1	2.3084 (19)	C33—C34	1.379 (11)
S1—P2	2.050 (3)	C33—H33	0.95
P1—C31	1.819 (7)	C34—C35	1.372 (12)
P1—C21	1.821 (6)	C34—H34	0.95
P1—C11	1.822 (7)	C35—C36	1.377 (11)
P2—O1	1.503 (6)	C35—H35	0.95
P2—O2	1.541 (5)	C36—H36	0.95
P2—C41	1.786 (9)	C41—C45	1.416 (10)
O2—H1	1.11 (11)	C41—C42	1.424 (11)
C11—C16	1.384 (10)	C42—C43	1.428 (11)
C11—C12	1.387 (10)	C42—H42	0.95
C12—C13	1.379 (11)	C43—C44	1.416 (11)
C12—H12	0.95	C43—H43	0.95
C13—C14	1.383 (12)	C44—C45	1.424 (11)
C13—H13	0.95	C44—H44	0.95
C14—C15	1.402 (13)	C45—H45	0.95
C14—H14	0.95	C51—C55	1.403 (13)
C15—C16	1.361 (12)	C51—C52	1.446 (13)
C15—H15	0.95	C51—H51	0.95
C16—H16	0.95	C52—C53	1.405 (14)
C21—C22	1.372 (10)	C52—H52	0.95
C21—C26	1.402 (10)	C53—C54	1.382 (15)
C22—C23	1.375 (12)	C53—H53	0.95
C22—H22	0.95	C54—C55	1.406 (13)
C23—C24	1.378 (12)	C54—H54	0.95
C23—H23	0.95	C55—H55	0.95
C24—C25	1.381 (11)	C1A—Cl2A	1.642 (17)
C24—H24	0.95	C1A—Cl1A	1.645 (17)
C25—C26	1.383 (10)	C1A—H1A1	0.99
C25—H25	0.95	C1A—H1A2	0.99
C26—H26	0.95	C1B—Cl2B	1.629 (14)
C31—C32	1.381 (10)	C1B—Cl1B	1.656 (14)
C31—C36	1.396 (10)	C1B—H1B1	0.99
C32—C33	1.399 (11)	C1B—H1B2	0.99
			
P1—Au1—S1	176.81 (6)	C33—C32—H32	120.1
P2—S1—Au1	99.14 (9)	C34—C33—C32	119.7 (8)
C31—P1—C21	106.3 (3)	C34—C33—H33	120.2
C31—P1—C11	104.6 (3)	C32—C33—H33	120.2
C21—P1—C11	106.1 (3)	C35—C34—C33	120.7 (7)
C31—P1—Au1	113.8 (2)	C35—C34—H34	119.6
C21—P1—Au1	113.1 (2)	C33—C34—H34	119.6
C11—P1—Au1	112.3 (2)	C34—C35—C36	119.8 (8)
O1—P2—O2	107.6 (3)	C34—C35—H35	120.1
O1—P2—C41	110.8 (3)	C36—C35—H35	120.1
O2—P2—C41	110.1 (3)	C35—C36—C31	120.5 (7)
O1—P2—S1	113.9 (3)	C35—C36—H36	119.7
O2—P2—S1	106.0 (2)	C31—C36—H36	119.7
C41—P2—S1	108.3 (3)	C45—C41—C42	107.2 (7)
P2—O2—H1	115 (5)	C45—C41—P2	126.0 (5)
C16—C11—C12	118.7 (7)	C42—C41—P2	126.8 (6)
C16—C11—P1	118.3 (6)	C41—C42—C43	108.6 (7)
C12—C11—P1	122.8 (5)	C41—C42—H42	125.7
C13—C12—C11	119.9 (7)	C43—C42—H42	125.7
C13—C12—H12	120.1	C44—C43—C42	107.5 (7)
C11—C12—H12	120.1	C44—C43—H43	126.3
C12—C13—C14	121.0 (8)	C42—C43—H43	126.3
C12—C13—H13	119.5	C43—C44—C45	108.1 (7)
C14—C13—H13	119.5	C43—C44—H44	125.9
C13—C14—C15	119.0 (8)	C45—C44—H44	125.9
C13—C14—H14	120.5	C41—C45—C44	108.6 (6)
C15—C14—H14	120.5	C41—C45—H45	125.7
C16—C15—C14	119.2 (8)	C44—C45—H45	125.7
C16—C15—H15	120.4	C55—C51—C52	106.9 (8)
C14—C15—H15	120.4	C55—C51—H51	126.5
C15—C16—C11	122.1 (8)	C52—C51—H51	126.5
C15—C16—H16	119	C53—C52—C51	106.9 (10)
C11—C16—H16	119	C53—C52—H52	126.5
C22—C21—C26	118.9 (6)	C51—C52—H52	126.5
C22—C21—P1	120.9 (6)	C54—C53—C52	109.1 (9)
C26—C21—P1	119.9 (5)	C54—C53—H53	125.5
C21—C22—C23	121.7 (8)	C52—C53—H53	125.5
C21—C22—H22	119.2	C53—C54—C55	108.6 (8)
C23—C22—H22	119.2	C53—C54—H54	125.7
C22—C23—C24	118.9 (7)	C55—C54—H54	125.7
C22—C23—H23	120.6	C51—C55—C54	108.5 (9)
C24—C23—H23	120.6	C51—C55—H55	125.8
C23—C24—C25	121.1 (7)	C54—C55—H55	125.8
C23—C24—H24	119.4	Cl2A—C1A—Cl1A	128 (3)
C25—C24—H24	119.4	Cl2A—C1A—H1A1	105.3
C24—C25—C26	119.4 (8)	Cl1A—C1A—H1A1	105.3
C24—C25—H25	120.3	Cl2A—C1A—H1A2	105.3
C26—C25—H25	120.3	Cl1A—C1A—H1A2	105.3
C25—C26—C21	120.0 (7)	H1A1—C1A—H1A2	106
C25—C26—H26	120	Cl2B—C1B—Cl1B	118.3 (16)
C21—C26—H26	120	Cl2B—C1B—H1B1	107.7
C32—C31—C36	119.4 (7)	Cl1B—C1B—H1B1	107.7
C32—C31—P1	121.9 (5)	Cl2B—C1B—H1B2	107.7
C36—C31—P1	118.5 (5)	Cl1B—C1B—H1B2	107.7
C31—C32—C33	119.8 (7)	H1B1—C1B—H1B2	107.1
C31—C32—H32	120.1		
			
Au1—S1—P2—O1	76.2 (3)	C11—P1—C31—C32	68.3 (7)
Au1—S1—P2—O2	−165.7 (2)	Au1—P1—C31—C32	−168.8 (6)
Au1—S1—P2—C41	−47.6 (2)	C21—P1—C31—C36	142.1 (6)
C31—P1—C11—C16	97.8 (6)	C11—P1—C31—C36	−105.9 (6)
C21—P1—C11—C16	−150.0 (6)	Au1—P1—C31—C36	17.0 (7)
Au1—P1—C11—C16	−26.0 (6)	C36—C31—C32—C33	−2.8 (12)
C31—P1—C11—C12	−76.7 (7)	P1—C31—C32—C33	−176.9 (6)
C21—P1—C11—C12	35.4 (7)	C31—C32—C33—C34	2.4 (13)
Au1—P1—C11—C12	159.5 (5)	C32—C33—C34—C35	−1.7 (13)
C16—C11—C12—C13	0.2 (11)	C33—C34—C35—C36	1.3 (14)
P1—C11—C12—C13	174.7 (6)	C34—C35—C36—C31	−1.6 (13)
C11—C12—C13—C14	0.2 (12)	C32—C31—C36—C35	2.4 (12)
C12—C13—C14—C15	−1.9 (12)	P1—C31—C36—C35	176.8 (7)
C13—C14—C15—C16	3.2 (12)	O1—P2—C41—C45	170.2 (6)
C14—C15—C16—C11	−2.9 (13)	O2—P2—C41—C45	51.3 (7)
C12—C11—C16—C15	1.2 (11)	S1—P2—C41—C45	−64.2 (7)
P1—C11—C16—C15	−173.5 (7)	O1—P2—C41—C42	−9.0 (8)
C31—P1—C21—C22	150.7 (6)	O2—P2—C41—C42	−127.9 (6)
C11—P1—C21—C22	39.8 (7)	S1—P2—C41—C42	116.6 (6)
Au1—P1—C21—C22	−83.8 (7)	C45—C41—C42—C43	−0.2 (8)
C31—P1—C21—C26	−35.8 (7)	P2—C41—C42—C43	179.1 (6)
C11—P1—C21—C26	−146.7 (6)	C41—C42—C43—C44	−0.1 (8)
Au1—P1—C21—C26	89.7 (6)	C42—C43—C44—C45	0.3 (8)
C26—C21—C22—C23	2.1 (12)	C42—C41—C45—C44	0.4 (8)
P1—C21—C22—C23	175.7 (7)	P2—C41—C45—C44	−178.9 (5)
C21—C22—C23—C24	−1.4 (13)	C43—C44—C45—C41	−0.4 (8)
C22—C23—C24—C25	−0.7 (13)	C55—C51—C52—C53	2.3 (10)
C23—C24—C25—C26	2.0 (13)	C51—C52—C53—C54	−1.9 (10)
C24—C25—C26—C21	−1.3 (12)	C52—C53—C54—C55	0.7 (10)
C22—C21—C26—C25	−0.8 (11)	C52—C51—C55—C54	−1.9 (9)
P1—C21—C26—C25	−174.4 (6)	C53—C54—C55—C51	0.7 (10)
C21—P1—C31—C32	−43.7 (8)		

### Hydrogen-bond geometry (Å, °)

**Table:**

*D*—H···*A*	*D*—H	H···*A*	*D*···*A*	*D*—H···*A*
O2—H1···O1^i^	1.11 (11)	1.33 (11)	2.432 (7)	173 (9)

Symmetry codes: (i) −*x*, *y*+1/2, −*z*+3/2.
